# Rare cranial osteopathy in a Northern Bottlenose Whale (*Hyperoodon ampullatus*) stranded in Rhode Island, Northwest Atlantic, in the 19th century

**DOI:** 10.29374/2527-2179.bjvm000426

**Published:** 2026-06-26

**Authors:** Marie-Francoise Van Bressem, Jennifer Anné, Koen Van Waerebeek

**Affiliations:** 1 Peruvian Centre for Cetacean Research, Museo de Delfines, Pucusana, Lima, Peru.; 2 ProDelphinus, Miraflores, Lima, Peru.; 3 Paleontology, North Carolina Museum of Natural Sciences, Raleigh, North Carolina, United States of America

**Keywords:** Ziphiidae, bony outgrowth, skull, North Atlantic, Ziphiidae, excreção óssea, crânio, Atlântico Norte

## Abstract

Few reports exist on the pathologies of the northern bottlenose whale (*Hyperoodon ampullatus*) and other Ziphiidae. We here describe a large bony outgrowth (exostosis) in the right pterygoid bone of an adult female *H. ampullatus,* ANSP 3004, that stranded on the US Atlantic coast in 1867. Images of the skull and 14 cranial measurements were examined and compared with two ‘normal’ adult female *H. ampullatus* specimens. The form, structure, and width of the right pterygoid were found to be abnormal. Its distal extremity was curved in a labial direction, while its proximal extremity was deformed by a large, bulbous bony outgrowth that partially obstructed the right nasal passage. Lytic lesions in the posterior end of the exostosis exposed deep resorption lacunae. The left pterygoid was thin and laterally displaced. No diagnosis was possible for lack of histological examination but the bony outgrowth was suggestive of an osteoma, a benign bone tumor commonly seen on the jaws and nasal sinuses of horses, bovines and sheep. The partial nasal passage obstruction may hypothetically have affected respiration to some degree, but since the female was lactating, general fitness appeared sufficient.

## Resumo

Existem poucos relatos sobre as patologias da baleia-de-bico-curto (*Hyperoodon ampullatus*) e outros Ziphiidae. Descrevemos aqui uma grande excrescência óssea (exostose) no osso pterigóide direito de uma fêmea adulta de *H. ampullatus* (ANSP 3004) que encalhou na costa atlântica dos EUA em 1867. Imagens do crânio e 14 medidas cranianas foram examinadas e comparadas com duas amostras «normais» de fêmeas adultas de *H. ampullatus*. A forma, a estrutura e a largura do pterigóide direito foram consideradas anormais. A extremidade distal estava curvada labialmente, enquanto a proximal estava deformada por uma grande exostose bulbosa que obstruía parcialmente a passagem nasal direita. Lesões líticas na extremidade posterior da exostose expuseram lacunas profundas de reabsorção óssea. O pterigóide esquerdo era fino e deslocado lateralmente. Não foi possível fazer um diagnóstico devido à ausência de exame histológico, mas a protuberância óssea sugeria osteoma, um tumor ósseo benigno comumente observado nas mandíbulas e seios nasais de bovinos. A obstrução parcial da passagem nasal pode, hipoteticamente, ter afetado a respiração até certo ponto, mas, como a fêmea estava a amamentar, a sua condição física geral parecia satisfatória.

## Introduction

Northern bottlenose whales *Hyperoodon ampullatus* (Forster 1770) are large-sized beaked whales (up to 9.7m) that inhabit deep, subarctic to temperate waters of the North Atlantic Ocean (south to ca. 37 °N) where they primarily feed on squid species of the genus *Gonatus* (Moors-Murphy, 2018; Whitehead et al., 2003, 2021). Bottlenose whales were heavily hunted throughout their range from 1880 to 1920 and again from 1937 to 1973, causing an estimated 40% reduction in their population (Taylor et al., 2008; Whitehead & Hooker, 2012). Although precise estimates are unavailable, the entire North Atlantic population is estimated to number at least 20,000 individuals. Currently, anthropogenic sound from military mid-frequency sonar and seismic surveys as well as bycatch in fisheries are the main threats for this species (Whitehead & Hooker, 2012). As a result of the historical depletion, the uncertainty of abundance estimates, and the exposition to harmful anthropogenic factors, northern bottlenose whales have been listed as ‘near threatened’ on the IUCN Red List of Threatened Species (Whitehead et al., 2021).

There are only few reports on the pathologies of *H. ampullatus*, even in stranded individuals. Dagleish et al. (2007) described severe mycotic encephalitis caused by *Aspergillus fumigatus* in a juvenile male *H. ampullatus* stranded in October 2006 at North Kessock, Scotland. Tattoo skin disease, a cetacean poxvirus infection, was also observed in this individual (Dagleish et al., 2008) as well as in a juvenile female that died in the Thames River in 2006 (Van Bressem et al., 2009). Tomilin (1967) reported that the ‘upper parts of the epidermis of *H. ampullatus* are sometimes affected by a pathological process of mycotic nature, resulting in the appearance of large white patches on the skin’. Feyrer et al. (2021) described anthropogenic marks, including probable entanglement and propeller-vessel strike scars, in 6.6% of the Endangered Scotian Shelf population (Nova Scotia). The skeleton of a young northern bottlenose whale from the Shetland Islands, curated at the Royal Belgian Institute of Natural Sciences at Brussels (RBINS), shows exostosis of the spiny processes of the caudal vertebrae. Another skeleton, at Naturalis Biodiversity Center, Leiden, is reported to have a ‘bony growth on the rib’ (Slijper, 1938; Tomilin, 1967). Slijper (1938), possibly referring to the RBINS specimen, also mentioned spondylitis deformans in this species, while Kompanje (1999) reported discarthrosis and zygarthrosis without further details. Knowledge of osteopathology in southern bottlenose whales *Hyperoodon planifrons*Flower (1882) is even more restricted (Barcellos, 1977).

In this context, it is important to promote studies of pathologies in beaked whales as to better understand the natural factors that may negatively impact their conservation. During a revision of cetacean specimens kept at the Academy of Natural Sciences of the Drexel University of Philadelphia, one of us (JA) observed a large, bulbous bony outgrowth i.e. an exostosis or "benign new growth from a bone surface" (Blood & Studdert, 1999) in the right pterygoid of an adult female *H. ampullatus* (specimen ANSP 3004) that had washed ashore on the US Atlantic coast in the 19th century. Here, we describe the case which represents the first record of this type of pathology in ziphiids. A potential diagnosis is discussed in the corresponding section.

## Case report

The 823 cm female *H. ampullatus* stranded with her calf on the shore of Narrangansett Bay near Tiverton, Newport County, Rhode Island, USA, in February 1867, after being hunted and probably harpooned, based on images associated with the description (Cope, 1869; Mitchell & Kozicki, 1975). Cope (1869) provided the following information on the adult specimen ‘*Its most striking feature is the relatively longer and more slender beak, and less elevated and prominent front. Thus, in the Newport specimen, it is one-twelfth of the* [body] *length, or 2 1/4 feet: equal three-fourths the distance between the eye and the spout hole. The prominent swollen front is in the Newport whale, considerably compressed, and the eye is placed in a strong longitudinal prominence on each side of the head*’. Cope (1869) further mentioned that the ‘muzzle’ [rostrum] of the female was longer than represented for European specimens, but this has not been confirmed. The skull was collected and donated to the museum, where it was entered as ANSP 3004, two years after collection, i.e., in 1869.

Images of the ANSP 3004 skull were examined by the authors and compared to ‘normal’ female *H. ampullatus* specimens kept at the Naturalis Biodiversity Center, Leiden, Netherlands (courtesy P. Kamminga), at the Museum of Comparative Zoology Mammalogy, Harvard University (MCZ; courtesy M. Omura), and the Royal Belgian Institute of Natural Sciences (RBINS; courtesy O. Lambert). Fourteen cranial measurements were taken (Table 1) and compared with unpublished measurements for two other adult, female specimens: MCZ- 25362, stranded in Beverly Farms, Massachusetts, in October 1923, and RBINS-1503, stranded in Zeeland, The Netherlands, in September 1840. Craniometric measurements were taken by different observers, so inter-observer variability may be significant. Surface scans of ANSP 3004 were made with a Faro Design Arm 1.0 scanner at 75µm resolution. Due to the size of the specimen, several scans were realized and stitched together using the 3D Systems Geomagic 2017 software to make a single surface model.

**Table 1:** Cranial measurements of northern bottlenose whale *Hyperoodon ampullatus*.

**Measurement Descriptions**	**ANSP 3004 Adult Female**	**%CBL**	**MCZ 25361 Adult Female**	**%CBL**	**RBINS 1503 Adult Female**	**%CBL**
Condylobasal length (CBL)	133.1	100.0	96.1	100.0	142.0	100.0
Greatest length of rostrum	83.9	63.0	53.8	56.0	93.0	65.5
Length, tip rostrum to margin superior nares	100.4	79.9	72.4	75.3	98.0	69.0
Width of rostrum in apices of antorbital notches	36.6	27.5	24.0	25.0	35.3	24.9
Greatest breadth of skull at preorbital process	68.2	51.3	49.2	51.2	55.0	38.7
Greatest breadth of skull at postorbital process	66.5	50.0	53.7	55.9	68.0	47.9
Greatest breadth of nasal bones	28.1	21.1	23.7	24.7	27.5	19.4
Greatest breadth of occipital condyles	26.0	19.5	20.2	21.1	23.2	16.3
Height of skull, maxillary crests to ventral point on pterygoids	56.4	42.3	40.5	42.1	52.0	36.6
Height of skull, vertex to most ventral on pterygoids	50.5	38.0	41.8	43.5	55.0	38.7
Length orbit on frontal	16.4	12.3	13.5	14.5	NA	NA
Length right pterygoid	29.7	22.3	29.2	30.4	NA	NA
Width right pterygoid	15.9	11.9	8.5	8.8	NA	NA
Length left pterygoid	27.2	20.4	29.3	30.5	NA	NA
Width left pterygoid	3.4	2.5	9.4	9.8	NA	NA
Largest width both pterygoids	18.7	14.1	17.6	18.3	NA	NA
Greatest breadth of skull across zygomatic processes of squamosals	62.6	47.1	49	51	NA	NA
Tip of rostrum to most posterior wing of pterygoid	101.9	76.6	73.2	76.2	NA	NA
Tip of rostrum to most anterior extension of pterygoid	73.1	54.9	45.2	47	NA	NA

Note: Cranial measurements of female *Hyperoodon ampullatus* ANSP-3004 compared to two mature females (MCZ-25361 & RBINS-1503). NA= not available. Measurements are in cm.

**Figure 1:**
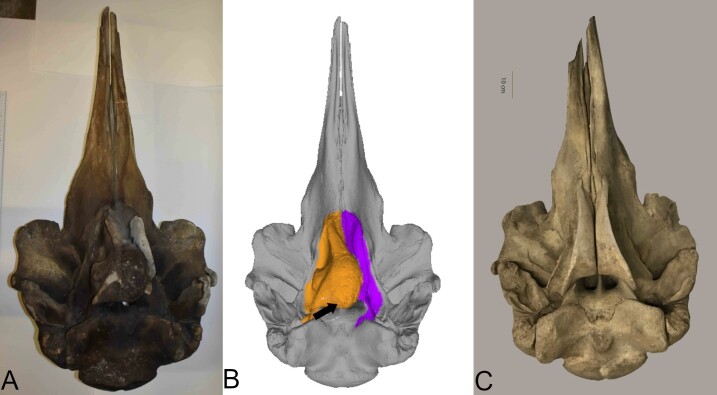
Ventral view of northern bottlenose whale *Hyperoodon ampullatus* skulls.

Note: (A) Ventral view of study specimen *Hyperoodon ampullatus* ANSP 3004; (B) surface model of this specimen and (C) in comparison with a ventral view of the normal skull of adult female *H. ampullatus* (MCZ 25362) from the Museum of Comparative Zoology, Mammalogy Department, Harvard University.

**Figure 2:**
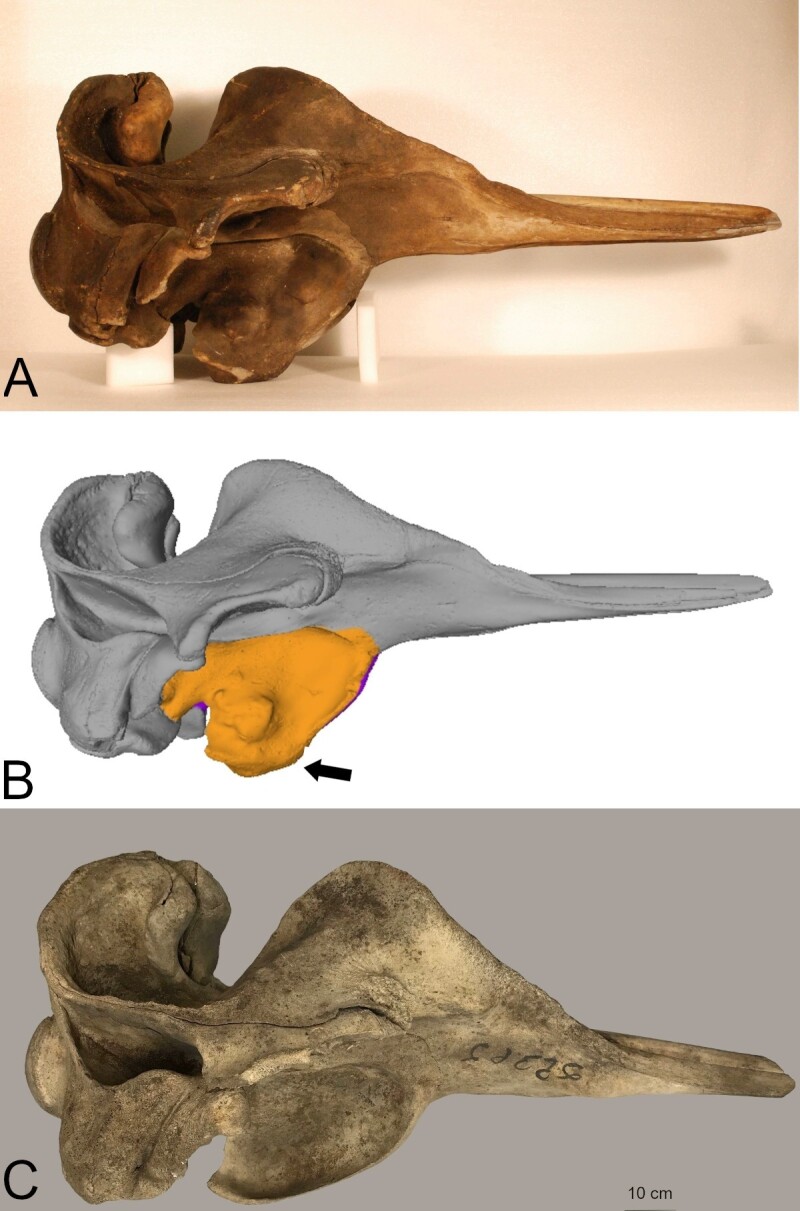
Right lateral view of northern bottlenose whale *Hyperoodon ampullatus* skulls.

Note: (A) Right lateral view of study specimen northern bottlenose whale *Hyperoodon ampullatus* ANSP 3004; (B) single surface scan model of this specimen showing a bony outgrowth in the right pterygoid bone (orange); (C) lateral view of an adult female *H. ampullatus* (MCZ 25362) with normal pterygoids at the Museum of Comparative Zoology, Mammalogy, Harvard University.

**Figure 3:**
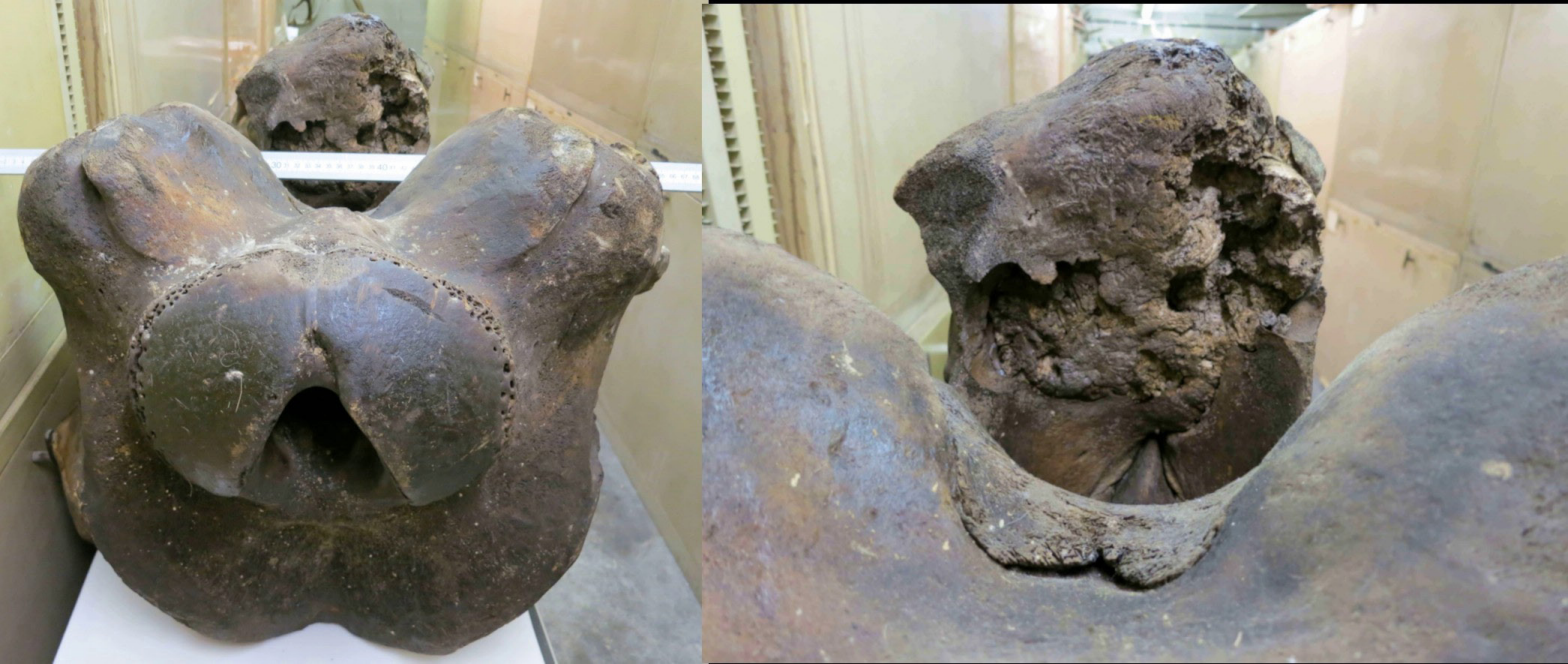
Osteopathy of the right pterygoid bone in *Hyperoodon ampullatus*.

Note: Osteopathy of the right pterygoid bone in *Hyperoodon ampullatus* ANSP 3004: (Left) Occipital view of the upturned skull with (anteriad) the bulbous exostosis seen protruding from the right pterygoid; (Right) close-up view of the osseous outgrowth exposing resorption lacunae, apparently from osteolysis.

The condylobasal length (CBL) of ANSP 3004 was 133.1 cm. Other measurements are provided in Table 1. Overall, the cranial dimensions of ANSP 3004, measured as % of CBL, were comparable to normal *H. ampullatus* skulls without osteopathy (see Table 1), with the exception of the pterygoid bones. Values for, respectively, ANSP 3004 and MCZ 25362, both adult females, were (in % CBL): length of left pterygoid (27.2 vs. 29.3), length of right pterygoid (29.7 vs. 29.2), and width of left pterygoid (3.4 vs 9.4). Only the width of the right pterygoid (15.9 vs. 8.5) was greater in ANSP 3004 due to the deformation. The form and structure of the right pterygoid were abnormal (Figures 1 and 2). Its distal (palatinal) extremity was strongly curved in a labial direction while its proximal (ventral-most) extremity was deformed by a large (143 x 125 mm), bulbous exostosis that extended laterally and medially, occluding the space between the pterygoids and partially obstructing the right nasal passage (Figures 1 & 3). There was also evidence of osteolysis exposing deep resorption lacunae in the posterior end of the exostosis (Figure 3). The left pterygoid was thin (34 mm), and slightly displaced laterally (Figure 1).

On the 3D model, much of the space of the right pterygoid sinus appeared filled with osseous tissue, while the left pterygoid sinus was unobstructed (Figure 1B). To what degree adjacent cranial bones, i.e., vomer, mesethmoid, ectecmoids, sphenoid, and (right) palatine, were affected could not be determined without (destructive) histology and hands-on examination by all authors.

Based on available observations, ANSP 3004 presented a marked ventral cranial malformation linked to a severe bulbous bony outgrowth enveloping the right pterygoid, of unknown etiology.

## Discussion

Lesions of the skull are poorly documented in the Ziphiidae family, with only one brief mention of osteolysis of the rostrum tip (premaxillary-maxillary bones) in an adult male Peruvian or lesser beaked whale, *Mesoplodon peruvianus*Reyes et al. (1991) stranded in Peru and curated at the Museo de Delfines, Lima, as MJE-001 (Montes et al., 2004; Reyes & Van Waerebeek, 2018). The case described here represents the first record of an osteopathy of the pterygoids in ziphiids.

Nematodes of the genus Crassicauda commonly infest the cranial sinuses of several Delphinidae species and cause trabecular or basket-like lytic lesions in the pterygoids, referred to as crassicaudiasis (Raga et al., 1982; Van Bressem et al., 2020). However, the pathology observed in ANSP-3004 bears no resemblance to those. Besides, these helminth parasites have never been reported in the cranial sinuses of Ziphiidae, although they have been found in their vascular and genito-urinary systems (Díaz-Delgado et al., 2016; Suárez-González et al., 2026; Tajima et al., 2015).

The bony outgrowth/mass documented in the right pterygoid of ANSP 3004 is suggestive of an osteoma, a benign, dense, clearly defined, slow-growing tumor consisting of well-differentiated bone tissue (Sato et al., 2017). In veterinary medicine, osteomas are most commonly seen in the jaws, nasal sinuses, and skulls of horses, cattle, and sheep, and are also found in dogs and cats (Blood & Studdert, 1999; Sato et al., 2017). Chronic osteitis following parasitic, fungal, or bacterial sinusitis (Belda et al., 2018; Dagleish et al. 2007), or a combination thereof, may have contributed to the observed osteopathy but is not thought to be the primary etiology. As destructive sampling and histologic analysis of the exostosis was not permitted on this intangible, historical specimen, the etiology of this skull lesion could not be confirmed.

The thick and robust pterygoid bones of beaked whales enclose large sinuses, each approximately a liter in volume in *H. ampullatus* (Scholander, 1940), lined with a rich venous plexus that play an important role in deep diving (Costidis & Rommel, 2012; Rommel et al., 2006). The malformation, including the severe bulbous exostosis in the right pterygoid, a partial obstruction of the right internal nare as well as a reduction of the left pterygoid width may hypothetically have affected respiration and diving to some degree. However, the impact of this osteopathy on deep diving, and thus on foraging (Rommel et al., 2006), still appeared inconsequential at the time of capture, given that the female was able to nurse a calf. No other data were available that would allow an assessment of the general health status of ANSP 3004.

While case studies as this one are very useful because yielding initial insights, what is needed are systematic future studies of diseases and traumas in representative samples of beaked whale skeletal material, available in many marine mammal collections. Such research should provide insights on the etiology, prevalence and severity of ziphiid osteopathies, and help elucidate individual and potential population impact.

The main limitations of the present study include the lack of histological analysis, the comparison with a limited number of individuals, and the potential variability associated with cranial measurements taken by different observers.

## References

[B001] Barcellos L. P. (1977). Nota sobre osteopatologia em um exemplar de *Hyperoodon planifrons* (Ziphiidae–Cetacea), Rio Grande do Sul, Brasil. Atlântica.

[B002] Belda B., Petrovitch N., Mathews K. G. (2018). *Sinonasal aspergillosis*: Outcome after topical treatment in dogs with cribriform plate lysis. Journal of Veterinary Internal Medicine.

[B003] Blood D. C., Studdert V. P. (1999). Saunders Comprehensive Veterinary Dictionary.

[B004] Cope E. D. (1869). Of the species of the cetaceans of the west coast of North America. Proceedings Academy of Natural Sciences of Philadelphia.

[B005] Costidis A., Rommel S. A. (2012). Vascularization of air sinuses and fat bodies in the head of the bottlenose dolphin (*Tursiops truncatus*): Morphological implications on physiology. Frontiers in Physiology.

[B006] Dagleish M. P., Barley J., Howie F. E., Reid R. J., Herman J., Foster G. (2007). Isolation of Brucella species from a diseased atlanto-occipital joint of an Atlantic white-sided dolphin (*Lagenorhynchus acutus*). The Veterinary Record.

[B007] Dagleish M., Foster G., Howie F., Reid R. J., Barley J. P. (2008). Fatal mycotic encephalitis caused by *Aspergillus fumigatus* in a northern bottlenose whale (*Hyperoodon ampullatus*). The Veterinary Record.

[B008] Díaz-Delgado J., Fernández A., Xuriach A., Sierra E., Bernaldo de Quirós Y., Mompeo B., Pérez L., Andrada M., Marigo J., Catão-Dias J. L., Groch K. R., Edwards J. F., Arbelo M. (2016). Verminous Arteritis Due to *Crassicauda* sp. in Cuvier’s Beaked Whales (*Ziphius cavirostris*). Veterinary Pathology.

[B009] Feyrer L. J., Stewart M., Yeung J., Soulier C., Whitehead H. (2021). Origin and Persistence of Markings in a LongTerm Photo-Identification Dataset Reveal the Threat of Entanglement for Endangered Northern Bottlenose Whales (*Hyperoodon ampullatus*). Frontiers in Marine Science.

[B010] Flower W. H. (1882). On the whales of the genus *Hyperoodon.*. Proceedings of the Zoological Society of London.

[B011] Forster J. R. (1770). Travels into North America: Containing its natural history, and a circumstantial account of its plantations and agriculture in general, with the civil, ecclesiastical and commercial state of the country, the manners of the inhabitants, and several curious and important remarks on various subjects.

[B012] Kompanje E. J. O. (1999). Considerations on the comparative pathology of the vertebrae in Mysticeti and Odontoceti; evidence for the occurrence of discarthrosis, zygarthrosis, infectious spondylitis and spondyloarthritis. Zoölogische Mededeelingen.

[B013] Mitchell E., Kozicki V. M. (1975). Autumn Stranding of a Northern Bottlenose Whale (*Hyperoodon ampullatus*) in the Bay of Fundy, Nova Scotia. Journal of the Fisheries Research Board of Canada.

[B014] Montes D. I., Chavera A. C., Van Bressem M.-F., Perales R. C., Falcón N. P., Van Waerebeek K. (2004). Descripción y evaluación anatómica de lesiones óseas cráneo-mandibulares en cetáceos odontocetos del mar peruano. Revista de Investigaciones Veterinarias del Perú.

[B015] Moors-Murphy H. B., Würsig B., Thewissen J. G. M., Kovacs K. M. (2018). Encyclopedia of marine mammals.

[B016] Raga J. A., Casinos A., Filella S., Raduan M. A. (1982). Notes on cetaceans of the Iberian Coasts. V. *Crassicauda grampicola* Johnston and Mawson, (1941 Nematoda), cause of injuries in the pterygoids of some specimens of *Grampus griseus.*. Säugetierkundliche Mitteilungen.

[B017] Reyes J. C., Van Waerebeek K. (2018). The lesser beaked whale *Mesoplodon peruvianus* Reyes, Mead & Van Waerebeek 1991 revisited, with biological observations on new specimens from Peru. Journal of Marine Biology & Oceanography.

[B018] Reyes J. C., Mead J. G., Van Waerebeek K. (1991). A new species of beaked whale *Mesoplodon peruvianus* sp.n. (Cetacea: Ziphiidae) from Peru. Marine Mammal Science.

[B019] Rommel S. A., Costidis A. M., Fernandez A., Jepson P. D., Pabst D. A., McLellan W. A., Houser D. S., Cranford T. W., van Helden A. L., Allen D. M., Barros N. B. (2006). Elements of beaked whale anatomy and diving physiology and some hypothetical causes of sonar-related stranding. The Journal of Cetacean Research and Management.

[B020] Sato R., Une Y., Madarame H., Hanami H., Kanai E., Murakami H., Tsukamoto A., Suzuki T., Ochiai H., Kikuchi M., Tanaka H., Onda K. (2017). A nasal osteoma with an acute course in a Japanese Black heifer. The Journal of Veterinary Medical Science.

[B021] Scholander P. F. (1940). Experimental investigations on the respiratory function in diving mammals and birds. Hvalrådets Skrifter.

[B022] Slijper E. J. (1938). Die Sammlung rezenter Cetacea des Musée Royal d’Histoire Naturelle de Belgique. Bulletin du Musée Royal d’Histoire Naturelle de Belgique.

[B023] Suárez-González Z., Fernández A., González J. F., Salgado-Jiménez N., Molpeceres-Diego I., Alonso-Almorox P., Sierra E. (2026). Pathological and epidemiological assessment of cranial crassicaudiasis in stranded cetaceans from the Canary Islands (1999-2024). Research in Veterinary Science.

[B024] Tajima Y., Maeda K., Yamada T. K. (2015). Pathological findings and probable causes of the death of Stejneger’s beaked whales (*Mesoplodon stejnegeri*) stranded in Japan from 1999 and 2011. The Journal of Veterinary Medical Science.

[B025] Taylor B. L., Baird R., Barlow J., Dawson S. M., Ford J., Mead J. G., Notarbartolo di Sciara G., Wade P., Pitman R. L. (2008). Hyperoodon ampullatus..

[B026] Tomilin A. G. (1967). Cetacea.

[B027] Van Bressem M. F., Duignan P., Raga J. A., Van Waerebeek K., Fraija-Fernández N., Plön S. (2020). Cranial crassicaudiasis in two coastal dolphin species from South Africa is predominantly a disease of immature individuals. Diseases of Aquatic Organisms.

[B028] Van Bressem M. F., Van Waerebeek K., Aznar F. J., Raga J. A., Jepson P. D., Duignan P., Deaville R., Flach L., Viddi F., Baker J. R., Di Beneditto A. P., Echegaray M., Genovo T., Reyes J., Felix F., Gaspar R., Ramos R., Peddemors V., Sanino G. P., Siebert U. (2009). Epidemiological pattern of tattoo skin disease: A potential general health indicator for cetaceans. Diseases of Aquatic Organisms.

[B029] Whitehead H., Hooker S. K. (2012). Uncertain status of the northern bottlenose whale *Hyperoodon ampullatus*: Population fragmentation, legacy of whaling and current threats. Endangered Species Research.

[B030] Whitehead H., Macleod C. D., Rodhouse P. (2003). Differences in niche breadth among some teuthivorous mesopelagic marine mammals. Marine Mammal Science.

[B031] Whitehead H., Reeves R., Feyrer L., Brownell R.L. (2021). Hyperoodon ampullatus.

